# AI-Optimized Technological Aspects of the Material Used in 3D Printing Processes for Selected Medical Applications

**DOI:** 10.3390/ma13235437

**Published:** 2020-11-29

**Authors:** Izabela Rojek, Dariusz Mikołajewski, Ewa Dostatni, Marek Macko

**Affiliations:** 1 Institute of Computer Science, Kazimierz Wielki University in Bydgoszcz, 85-064 Bydgoszcz, Poland; dmikolaj@ukw.edu.pl; 2 Faculty of Mechanical Engineering, Poznan University of Technology, 60-965 Poznan, Poland; ewa.dostatni@put.poznan.pl; 3 Department of Mechatronics, Kazimierz Wielki University in Bydgoszcz, 85-064 Bydgoszcz, Poland; mackomar@ukw.edu.pl

**Keywords:** 3D printing, exoskeleton, mechanical requirements, material properties, material selection, computational intelligence, artificial neural networks

## Abstract

While the intensity, complexity, and specificity of robotic exercise may be supported by patient-tailored three-dimensional (3D)-printed solutions, their performance can still be compromised by non-optimal combinations of technological parameters and material features. The main focus of this paper was the computational optimization of the 3D-printing process in terms of features and material selection in order to achieve the maximum tensile force of a hand exoskeleton component, based on artificial neural network (ANN) optimization supported by genetic algorithms (GA). The creation and 3D-printing of the selected component was achieved using Cura 0.1.5 software and 3D-printed using fused filament fabrication (FFF) technology. To optimize the material and process parameters we compared ten selected parameters of the two distinct printing materials (polylactic acid (PLA), PLA+) using ANN supported by GA built and trained in the MATLAB environment. To determine the maximum tensile force of the exoskeleton, samples were tested using an INSTRON 5966 universal testing machine. While the balance between the technical requirements and user safety constraints requires further analysis, the PLA-based 3D-printing parameters have been optimized. Additive manufacturing may support the successful printing of usable/functional exoskeleton components. The network indicated which material should be selected: Namely PLA+. AI-based optimization may play a key role in increasing the performance and safety of the final product and supporting constraint satisfaction in patient-tailored solutions.

## 1. Introduction

The use of robotic exoskeletons based on the principles of motor learning can constitute an important part of rehabilitation. It offers increased efficiency due to the generation of complex, controlled, multisensory stimulation of the patient, useful in modifying the plasticity of the neural connections through the experience of movement [1]. Innovative solutions in assistance robotics may be achieved thanks to both the analysis of patient-generated bioelectrical signals, artificial intelligence applied to their classification and control, and novel materials used, especially to personalize the features of the exoskeleton [2]. Constraint satisfaction in patient-tailored solutions is difficult to achieve due to the many factors influencing the ultimate results, which moreover change depending on patient health status, functional abilities, support requirements, and body dimensions [3]. Further studies on the technological aspects of the material involved in three-dimensional (3D) printing in the broad context are of significant importance in the development of patient-tailored rehabilitation devices. Personalized solutions are especially important in neurorehabilitation (in stroke survivors, patients after traumatic brain injury, spinal cord injury, etc.) and in care for elderly patients with neurodegenerative diseases (aMCI—amnestic Mild Cognitive Impairment), Alzheimer’s disease, Parkinson’s disease, etc.). Their number is increasing in developing and developed countries, and despite early diagnosis the intervention level and type of resulting dysfunction may significantly vary and thus require adaptive forms of therapy, rehabilitation, and care. There is a need to highlight continuously why such solutions are important, especially within interdisciplinary therapeutic teams incorporating such novel technologies as 3D printing and reverse engineering. Reverse engineering is defined here as scanning, examining, and analyzing, in detail, a body component, product, or device in order to discover the rules of production, usually with the aim of achieving a better fit (here, for example, an exoskeleton to the hand) or of producing something similar, often with improved properties (here, for example, from other material). Such solutions should also define their purpose and significance [3]. The limited number of specialists (including engineers) and relatively long duration of their education results in the current high cost of personalized therapy, suggesting the need for novel solutions, mostly technical [4]. There is a need for more advanced technologies, combining semi-automated (based on artificial/computational intelligence, respectively AI/CI) design systems, novel multi-material 3D printing technologies, and advanced materials with features that are easily adaptable to the needs of patients and the features of their bodies, their nervous systems, and their type and level of dysfunction [5]. A careful review of the current state of research shows that the majority of these assistive technologies have several limitations precluding their widespread use, including high energy demand, lengthy donning and doffing time, and poor movement patterns [6].

The most important hypothesis is that the intensity, complexity, and specificity of robotic exercise may be supported by patient-tailored 3D-printed solutions [7]. Three-dimensional printing, also called additive manufacturing, is characterized by the computer-controlled processes used to create 3D products (objects) with precise geometric shapes by depositing material(s), usually in layers in contrast to traditional manufacturing requiring machining or other ways of removing surplus material [8,9]. Their use is suitable for biomedical applications, although they still do not cover all possible clinical areas and associated requirements. There is a need for further research, development, and commercialization, including in the field of advanced technologically 3D-printed exoskeletons [10].

The method proposed by the authors introduces into the area of research an innovative approach to computational intelligence (CI) used in the rare subject of three-dimensional printed exoskeletons. There are only a few similar studies that have been carried out on the basis of artificial neural network (ANN), so the direct added value of this study compared to other researchers in the methods used may be its novelty and complexity.

### 1.1. Literature Review of Clinical Applications of 3D Printing

3D printing is a promising technology, with a number of novel biomedical applications being developed. It is regarded as a future alternative to the current clinical approach to preventive medicine, diagnosis, treatment, rehabilitation, and care. It has the potential to cause a technological revolution in materials engineering, especially in the area of patient-tailored therapies, including 3D printed orthoses and exoskeletons.

Typically, for an exoskeleton, the 3D architecture of the device is designed using computer-aided design (CAD) software, based both on 3D scans of the patient’s body parts and pre-programmed template(s), and then printed with a classic polylactic acid (PLA) filament [6] or antimicrobial 3D printing polymer to prevent skin infections during rehabilitation [11]. The details of the processes are much more complicated, of course [12], mainly due to the combination within one process of knowledge and experience from many scientific and engineering disciplines necessary to develop a customized (tailor-made) product. Therefore, while the main rules of the process are repeated, the product can be unique each time, as can be the material parameters of the printout. Our approach to a 3D printed hand exoskeleton is shown in [13] and is different from the existing methods; the main differences are due to the complexity of the whole procedure including:

- Assessment of the patient’s functional capabilities,
- identification and analysis of functional needs,
- required support from the exoskeleton,
- the implementation of these arrangements in the template of the exoskeleton,
- tests and amendments,
- transfer of exoskeleton operation from a controlled environment to the patient’s home environment [13].

The range and method of hand movements as well as the natural reflexes protecting the fingers of the hand (e.g., reversing when feeling heat or pressure) make interaction between the hand and the exoskeleton on the hand crucial when performing reflexes. The use of an exoskeleton in activities of daily living must not deprive the user of the natural reflexes that are necessary, e.g., when the hand’s function returns and hand is used without an exoskeleton or using another, less supportive model of hand exoskeleton. In this study effort has been made to check the risks that occur more frequently, i.e., when carrying or grasping objects. In this case, natural torsional forces are much less frequent (in patients) and are planned to be tested in subsequent stages of our hand exoskeleton research as part of a comprehensive approach.

Mechanical and manufacturing problems are complex, but, here, they are only part of a larger manufacturing process, so their automation would significantly speed up the patient’s supply of exoskeletons. Currently produced exoskeletons are too complicated, heavy, and expensive. Change in patient’s state of health makes it necessary to replace individual parts of the exoskeleton. In addition, significant proportion of users of such exoskeletons are children, who, as they grow and develop, require even more frequent replacement of the entire exoskeleton or parts of it, because regulations do not allow for all changes. Exoskeletons and other rehabilitation supplies are an opportunity for them to learn, work, and live independently.

Despite the rapid development of material engineering, the number of materials available for the 3D printing of medical devices and the associated possibilities of shaping their required mechanical and material characteristics are still limited by the following main 3D printing material features:

- Dimensional properties: Accuracy and volumetric shrinkage,
- production speed or (in smaller parts) number of production cycles per minute,
- mechanical properties,
- thermal properties,
- properties of chemicals and their ability to be used in direct contact with the human body [9].

Further research is necessary on the development of the current and novel generations of 3D printing materials dedicated primarily to biomedical applications in order to eventually meet all requirements, particularly:

- Required ranges of shaping the characteristics,
- very high repeatability of mechanical parameters,
- anti-allergic properties and biocompatibility,
- no changes in mechanical parameters in contact with living tissues,
- resistance to processes related to ageing under the influence of, for example, Ultraviolet (UV) radiation (not always fully known),
- resistance to moisture, body fluids, etc.,
- biodegradability,
- recyclability,
- possibility of certification as medical materials.

Some of the material aspects of orthoses have been introduced in papers by Górski et al. In their first paper, a two-part orthosis was printed based on a 3D scan of the patient’s upper limb, where each part was formed from different materials due to the need to fulfill different functions. The 3D printing was done as a single process without additional assembly operations, using double-head fused deposition modeling (FDM), one of the most widespread 3D printing technologies [14]. The second paper presented an algorithm for the optimization of FDM process parameters using finite element analysis (FEA), where the optimization influenced the characteristics of the final product, especially in terms of strength [15]. The model was created in an STL (stereolithography) file using a design program such as AutoCad. It was then imported into a printer program equipped with slicer function, which mathematically (in order of X, Y, and Z coordinates) divided the data from the STL file into horizontal layers, generating the required supports (if needed) and planning the paths required for the extrusion head. The filament (here: Acrylonitrile butadiene styrene (ABS) material) goes to the temperature-controlled FFF extruder head, where it was heated to a semi-fluid state. The extruder head and sets the material in layers on clutchless base, one layer at a time in X and Y coordinates. When the layer was ready, the head moves in the Z direction to the next layer. The layers were joined and fixed, and the printed object became a permanent three-dimensional printout.

Due to the almost 24 h, 7 days a week use of a hand exoskeleton, we are fully convinced that the safety precautions of a hand exoskeleton should be similar to those of gloves, hand orthoses, or elements of wheelchairs. Thus, similar restrictions concerning material use should be incorporated [16]. Unfortunately, the current studies on hand exoskeletons concerning the technological issues have taken into consideration only the design, simulation, and control of such devices, without paying particular attention to material requirements and restrictions [17]. However, recent developments may change this situation. A relatively novel solution comprises stimuli-responsive polymer systems—functional materials that can change their physical and/or chemical properties in response to external stimuli (mechanical stress, temperature, humidity, radiation, chemical agents, pH, and electric and magnetic fields). Such micro- and nanoscale structures adapted for use as artificial muscles, soft sensors and actuators, smart textiles, and soft robots would significantly increase the range of possibilities for the whole area of 3D-printed exoskeletons [18]. The aforementioned (r)evolution also concerns the use of silk fibroin (SF) based bioinks in 3D-printing applications, demonstrating unique physical properties, chemical components, and bioactivities [19]. A novel rubber composite features shape memory (i.e., shape recovery and fixity) with a tailorable transition temperature [20]. Soft robotic actuators developed with 3D-printing technology use polyelectrolyte hydrogels, but still require sophisticated computational models to estimate their behavior. Soft actuators containing Takagi-Sugeno fuzzy sets (T-S) are useful as voltage-controlled devices, reacting to a wide range of input voltage changes. Moreover, the proposed modeling method, after modification, works with other polymer systems with 3D imprint reacting to stimuli [21] (gelatin and interpenetrated polymers). The above-mentioned stimulus-responsive materials allow one to measure at the same time RH (Relative Humidity), furthermore, water vapor is used to change the film thickness and its refractive index [22]. Four-dimensional (4D) systems of stimulus-responsive polymers can be used as self-morphing structures, such as shape-memory polymers (self-bending/morphing/rolling). A simple computational tool (based on a commercial finite element (FE) software package, Abaqus) is required to understand and control their thermo-mechanical behaviors [23]. Synergy between the explained methods is needed to fully exploit opportunities for 3D printed patient-tailored medical devices. There are many new and emerging solutions that are hopeful. Their complexity requires the use of new CI methods in order to shorten the procedures for the development of final products and avoid possible erroneous paths.

There is no doubt also a need for more AI-based tools that can be implemented to engineer practical 3D-printed parts, including advanced shapes, moving parts, and self-morphing/rolling structures.

### 1.2. Aim of the Study

The main aim of the work was to computationally optimize the 3D-printing process in terms of features and material selection, to achieve the maximum tensile force of the exoskeleton sample based on artificial neural network optimization supported by genetic algorithms (GA). The use of neural network combined with genetic algorithm is an innovative approach to material selection, allowing the maximization of material strength. Another problem that these AI methods solve is the optimization of the parameters related to the 3D printing technological process. The authors have experience in using AI methods to solve technical problems (e.g., in creating classifier and prediction models [24,25] and in selecting materials in ecodesigning [26,27] in the form of decision trees and neural networks).

## 2. Materials and Methods

In achieving the ability to operate high technologies, it is essential not to ignore the need to educate the required technological specialists and to create an organization capable of managing the high level of production complexity. Modern economies are built around the so-called technology complex—a system of synergy between the competences and components of a complex solution. It is necessary to use concurrent design, i.e., the simultaneous creation of complementary solutions based on uniform architecture, and decentralized system of cooperation. The prerequisite for achieving these levels of ability is to break the communication barriers, to use management methods based on task mapping and the freedom of data exchange, and to establish common areas of cooperation within process management. If this fails, then it may significantly limit the transparency and understanding of the dependencies and hence productive team cooperation. CI solutions can provide an easier and quicker way to achieve specific properties of the printed object.

The current pandemic situation requires developed economics, involving the process of change and rapid application of innovations (technological and organizational), where both people and processes need to participate in this change preferably as team/community to feel that they play a part in transforming our way of life and approach to emerging technologies and associated novel processes. This can be done from practical point of view, for example, by responding directly to the needs of the public and healthcare professionals for 3D printed protection measures, reacting immediately to their comments, adapting more quickly to changing needs, and increasing the volume of products produced.

Process management optimizes processes toward the goals established by the strategy, through more efficient processes that provide increased and longer-lasting success. Process management is supported by clear process organization, responsibility for pending processes, and the motivation system:

1. Process identification (based on reference models or benchmarking)
  - top-down approach,
  - bottom-up approach.
2. Process modeling (diagnostic or prognostic approach)
  - manual,
  - computational (including AI/CI-based).
3. Process introduction
4. Process control (continuous or cyclic)
  - goal planning,
  - resource organization,
  - control,
  - motivation or stimulation (where available).

The aforementioned requirements for the hand exoskeleton fall very generally within the goal-oriented requirement engineering (GORE) paradigm (use of the various tools):

- Mechanical features should be combined with control techniques to follow the users’ movements transparently,
- the system must be able to reproduce human movements at functional level (a certain number of degrees of freedom to be controlled, etc.),
- intervening in a particular desired way with the subjects’ movement must be possible (control of the interaction force between user and robot),
- wearability,
- usability,
- low cost,
- other non-functional requirements aimed at improving user acceptance (see user experience methods and tools) [28].

Requirements concerning material and 3D-printing issues are only indirectly mentioned above.

A review of the main databases shows that 3124 scientific articles were published between 1963 and 2020, being focused on the topic of the construction and application of medical exoskeletons, while only 322 (10.31%) covered the issue of the hand exoskeleton [29]. There are very few studies concerning 3D-printed hand exoskeletons [13], all since 2015, but none have covered the issue of material [30].

We are aware that the parameters of materials used to build the exoskeleton may be the result of a compromise as an exoskeleton may have to fulfill many functions and ensure a suitable balance between weight, stiffness, dexterity, and safety [31], described by many mechanical and material parameters, such as the filling factor (constraint satisfaction effect). We selected a part of the exoskeleton to analyze its parameters and then optimized them to achieve better mechanical parameters, which were checked under laboratory conditions [12].

To create and 3D-print the selected part of the hand exoskeleton we used Cura 0.1.5 software (3D Ultimaker, Utrecht, The Netherlands) with SLICER and fused filament fabrication (FFF) technology. The 3D printers BCN Sigma (BCN3D, Barcelona, Spain) and MakerBot Replikator 2X (MakerBot, New York, NY, USA) were used as hand scanners, and a set of the sport cameras were used.

To determine the maximum tensile force of the exoskeleton samples, they were each mounted in the grips of an INSTRON 5966 universal testing machine (Instron, High Wycombe, UK).

The choices of material (between PLA and PLA+) and optimal process parameters (using ANN and GA) were made in the MATLAB environment (R2020a, MathWorks, Natick, MA, USA).

## 3. Procedures

Our optimization algorithm influenced the characteristics of the final product: An element of an exoskeleton, especially in terms of the strength and durability of the part, reflected in its further safe use (e.g., by children).

### 3.1. Material Factors

To create the exoskeleton, we used reverse engineering procedures based on 3D-scans of the patient’s hand and pre-programmed templates related to the exoskeleton. Such an approach offers the ability to quickly create or recreate objects despite their complex shapes and internal structures.

The main physical properties required of exoskeleton parts include durability, elasticity, hardness, compressibility, resilience, and response to temperature. Fifty printed samples of exoskeleton parts were chosen for the final assessment.

To create the part of the exoskeleton from a 3D-scan based CAD file (.STL, GCodes), FFF, also called fused deposition modeling, technology was used, with two kinds of 2.85 mm diameter filament: (PLA and PLA+); infill rate: 100%; and retraction: Enabled. The temperatures selected depended on the features of each filament (Table 1).

The values of the printing process parameters are usually chosen based on recommendations by the equipment manufacturer, scholarly papers, experience, and their implication and significance according to the pilot investigations. Thus, it was our intention to optimize and objectivize the process of parameter fitting using CI tools and methods.

We selected PLA, an aliphatic polyester of organic origin that is fully biodegradable, due to its well-known general characteristics:

- Slight material shrinkage,
- a heated bed is not necessary,
- good durability (but lower than in the case of ABS),
- higher production and processing costs,
- the possibility of deformation under the influence of high temperatures
- limited flexibility,
- relatively low printing temperature: 180–220 °C,
- insolubility,
- does not emit harmful fumes.

PLA is produced from natural raw materials and, due to its inferior physical properties, is used less frequently in industry, but more often in biomedical applications, including the production of dental implants, surgical threads, disposable packaging, dishes, and low-cost 3D printing. Moreover PLA-printed objects have a smoother, shiny surface with full color saturation, which may be important in everyday applications (external, visible orthoses, exoskeleton) as an attractive form may help avoid stigmatization, e.g., in children.

PLA must be stored in dry conditions, since moistened PLA, even after thorough drying, may lose its properties during 3D printing as the heating process causes reactions with any remaining water. These problems with PLA (for ABS) filaments concern places having a higher air humidity i.e., above 50% RH (relative humidity, air humidity). Water particles penetrate the material, causing it to swell and slightly enlarging the wire diameter. This may severely deteriorate the quality of the printed items, rendering them unusable. Drying of PLA (for ABS and nylon) is possible but risky, requiring a suitable container exposed to the sun or heated with a bulb and containing a moisture absorber. The material should also be protected against the problem of air pollution, e.g., dust particles, cat hair, or sanding dust settling on the filament.

There are commercially available filaments that offer the possibility of steam sterilization as well as antibacterial filaments that include copper particles.

In addition to the basic properties of PLA, the PLA+ filament is additionally characterized by:

- Increased tensile strength (by 8.5%),
- increased bending strength (by 34.40%),
- increased modulus of elasticity in bending (by 14%).

### 3.2. Optimization Procedures

**Figure 1:**
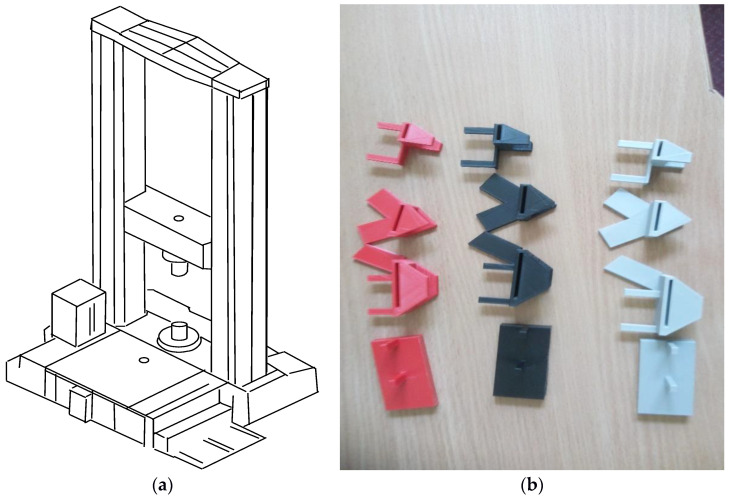
(**a**) INSTRON 5966 and (**b**) test samples of the exoskeleton parts.

3D-printing by FFF technology with selected types of filaments was carried out at a temperature of 22 ± 2 °C (these values were downloaded directly from the 3D printer software) and RH of 50 ± 10%. Fifty exoskeleton part samples were printed (Figure 1b).

The aim of the research was to determine the maximum tensile force of the exoskeleton samples under various material conditions. The tests consisted of mounting each sample in the grips of an INSTRON 5966 testing machine (Figure 1a) and subjecting them to monotonically increasing tensile load at piston velocity of 0.2 mm/s. The tests were carried out at temperature of 22 ± 2 °C and air humidity (RH) of 50 ± 10%. The registration test included the instantaneous values of the load and the displacement of the testing machine grip. The tests continued until the sample cracked and separated completely.

An artificial neural network (feed-forward neural network with back-propagation algorithm) written in MATLAB 16.0 was used for training and optimization purposes. These are often used to solve problems where the complete formulation is unknown (i.e., lack of model or mathematical representation). Several ANN models were built on the basis of three different configurations of input data and how they were defined. It was investigated how far it was possible to simplify the description in order to reduce the number of input variables and thus shorten the time necessary to build and train the network. The parameters were optimized more efficiently using ANN: While optimizing the number of input variables during the construction of the ANN model, a genetic algorithm was used to reduce the number of variables. Only 3D printing parameters included in Table 1 read directly from the same FFF printer’s control software were used. Conditions for the selection of input parameters were as follows:

- Their numerical values had to be homogeneous,
- the process of obtaining the above parameters (Table 1) from the 3D-printer software could not be time-consuming,
- they had to enable calculation of the required output variables of ANN (angle of rotation of the table and the head).

The learning and mapping abilities of ANN are often used to address the non-linear nature of 3D printing processes. Scaling the values of the input variables was performed during the construction of the ANN to maintain the uniformity of interpretation of all the signals within the network and the weights related to them. The ANN input variables were normalized, as the collected variables (Table 1) were characterized by a large and diverse range. This was done to prevent ANN input variables with very high values (e.g., in the range 10–100) to be considered by the network to be more significant than those with lower values (e.g., in the range 0–1).

Multiple ANN topologies were developed by researchers and reported in the literature, including feed-forward, recurrent, and functional links with varying levels of forecast precision.

The ANN structure is normally selected on the basis of:

- Developers’ prior knowledge,
- hit and miss trial approaches.

A genetic algorithm is a directed heuristic search technique that mimics the biological processes to perform a random search in a defined, N-dimensional set of possible solutions [19]. GA can seek and locate the best solution in a given search space (optimization problem), which it has proved very effective, including computational problems where solutions are multiple and non-deterministic.

GA allowed the automatic optimization of the ANN structure, since GA applies an original fitness function. According to the adopted fitness function, the networks were assessed after 1000 epochs. The error achieved after this number of epochs constituted one component of the evaluation, while the other was the penalty for the complexity of the web. To find the optimal ANN topology with genetic manipulation of chromosomes, the mean square error (MSE) of the model was used as the fitness function.

The fitness evaluation function was defined as:

F = MSE + (1 − α) × (w/w_n_) × 10^−5^ + α × (n/n_n_) × 10^−5^ (1)

where:

- MSE—mean square error of the neural network (NN) model,
- α—weight constant, which is set to 0.8,
- w—number of weighted interconnections between the input and the hidden layer,
- w_n_—maximum number of all interconnections,
- n—number of neurons in the network,
- n_n_—maximum number of neurons in network,
- 10^−5^—synaptic weight constant.

MSE after certain number of training epochs is calculated as:

(2) $$\mathrm{MSE}=\;\sum_{i=1}^{p}\frac{{\left|i_{f}-{\;i}_{a}\right|}^{2}}{p}$$

where:

- p is the number of samples used during the training processes,
- i_f_ and i_a_ are the forecasted output and the actual output.

The following parameters for GA were adopted:

- Population size: 15,
- number of iterations of the algorithm: 10,
- selection method: Tournament,
- maximum number of neurons in the layer: 70 [33].

**Figure 2:**
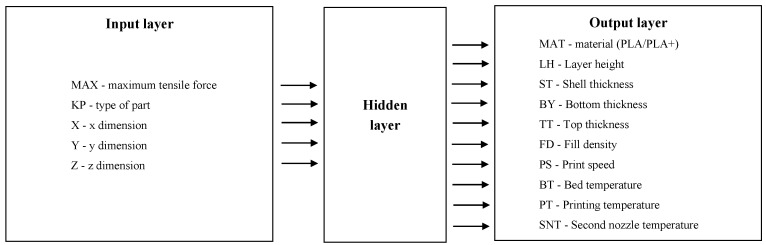
Artificial neural network (ANN) structure: Inputs and outputs.

We used a three-layer feed-forward neural network (Figure 2). The back-propagation algorithm (BP) was used, as the conventional and most popular gradient-based local search optimization technique. Optimization of the connection weights of the multi-layer perceptron (MLP) was set to the minimization of the error function (i.e., average mean square error between target and actual outputs averaged over all training examples). The neural network weights were presented (instead of setting the aforementioned scales to small random numbers) to avoid a slow error convergence rate and being trapped at local minima. We used naive initialization techniques rather than more advanced solutions (LeCun initialization, Xavier initialization, Kaiming initialization) due to the “nice region” being unknown. In commercial applications many techniques are used to achieve this in a semi-automatic way, such as artificial bee colony (ABC) and simulated annealing.

Initially, during the retrospective analysis of the parameters from 3D-printing, attempts were made to obtain as much information about the influence of 3D printing parameters as possible. In subsequent stages of the work, the information was selected for its impact on modeling the problem of dose distribution optimization by networks.

Each layer of the network contained neurons with the same activation function (Table 2). The models used the activation function of the sigmoidal type (the hyperbolic tangent) because it is a very flexible nonlinear, continuous, and differential function.

The number of inputs (n) and outputs (m), as well as the number of neurons in the hidden layer, was set subsequently, experimentally, to find the minimum required number of inputs and outputs (minimal workload). Tested ANNs based on the authors’ experience were collected in sets for the number of neurons and weight and structure of connections and then tested for the speed of learning, the speed of generalization of the result, and the minimization of the MSE. We should take into consideration that some parameters (Table 1) may change over a relatively narrow range due to the technical requirements of the whole processes or due to limitations created by the particular type of the material, 3D printer, and its software (e.g., ranges of adjustment). The number of neurons in the hidden layer depends on the values n and m, reflecting the generalization capabilities of the network.

**Figure 3:**
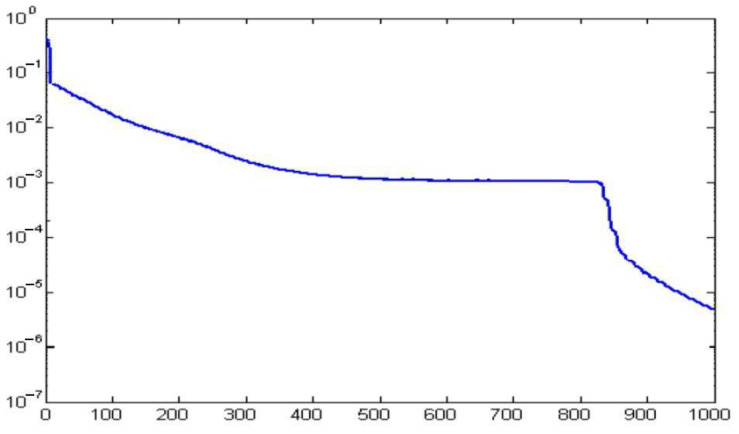
Values of mean square error (MSE) during learning.

The learning processes required multiple presentation of the learning patterns and modification of the weights. Each of the presented learning patterns took 10,000 iterations (repetitions) on average, until the network reached the assumed level of MSE. The number of learning epochs ranged from 500 to 1000 (Figure 3).

**Figure 4:**
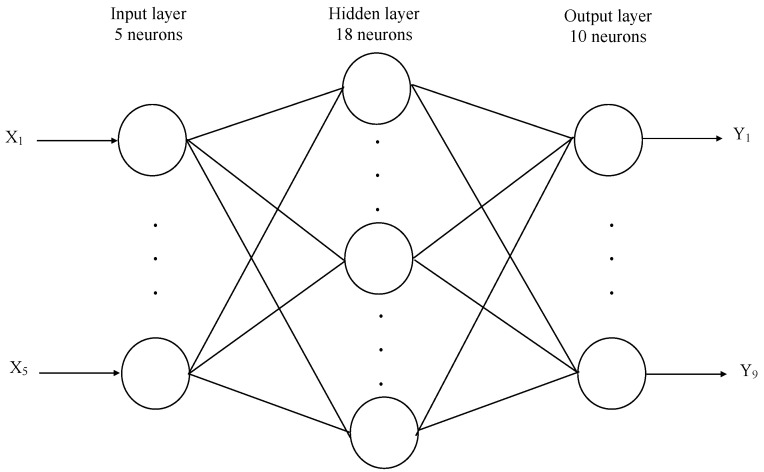
Structure of the optimal ANN.

The problem of optimizing the ANN structure is a key question for designers. An MLP type of network performs the mapping of an n-dimensional vector into an m-dimensional input signal. Such an approximation is possible using one hidden layer with 2n + 1 neurons, although the number of neurons in the hidden layer usually varies between n and 3n (Table 2, Figure 4, MLP 5-18-10 is the best).

The proposed method of optimizing constitutes a step towards the standardization of the problem of selecting the 3D printing parameters within Industry 4.0 through the use of AI methods.

## 4. Results

Manufacturing suitable ready-to-use exoskeleton parts based on FFF is a challenging task. As we mentioned above, a key condition for our study replication may constitute suitable PLA or PLA+ material choice, storage, and preparation, and the same procedures as for the 3D-printed objects.

Prior to 3D printing, slicing software determines how to break the 3D digital model into the layers required for printing by the FFF printer. In order to print a particular pattern:

- The nozzle (or the print bed or both—depending on the type of printer) moves while the plastic is being extruded,
- a simultaneously heated nozzle which ejects molten plastic, depositing it in thin layers, one on top of another, initially onto the print bed, layer-by-layer forming the whole 3D printed part,
- the filament layers that are fused together, due to the thermal fusion bonding that occurs between individual layers, to create a solid object [34].

Manufacturing processes parameters not mentioned in Table 3, along with the configuration and filament color (grey in both PLA and PLA+), did not affect the assessed printed part properties significantly.

**Figure 5:**
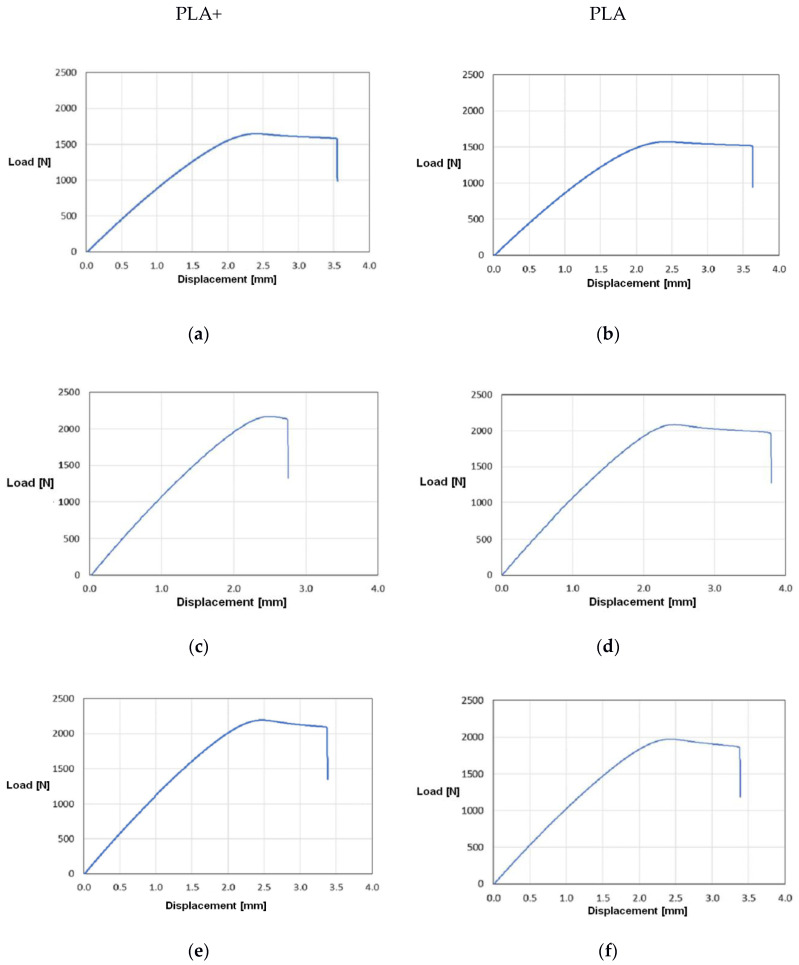
Tensile force of the selected exoskeleton sample under various material conditions: PLA+: Weak part (**a**), optimal part (**c**), strong but heavy part (**e**), PLA: Weak part (**b**), optimal part (**d**), and strong but heavy part (**f**).

ANN was used to optimize the tensile load, which was then validated experimentally. The results for selected samples of PLA and PLA+, including optima for PLA+, are shown in Figure 5a–f and Table 3. The results shown in Figure 5 are better for PLA+ than for PLA, which was definitely due to its greater elasticity. Thus, the selection of PLA and PLA+ depends on the required stiffness of the entire exoskeleton part and should be taken into account in the exoskeleton project.

Balancing the technical requirements with user safety constraints requires further analysis to advance the project from the initial stages. Issues concerning materials are very important in the construction and usefulness of an exoskeleton, especially a 3D-printed exoskeleton. It requires achieving a balance between many exoskeleton features, including those reflecting the patient’s body features. AI-supported optimization may play a key role in the paper, increasing the safety and durability of the final product.

The maximum tensile force for selected exoskeleton samples was approximately 8–9% lower for parts made from PLA than for those made of PLA+, compared to the 8.5% declared by the producer, but the optimal part compromised between maximum tensile force and low weight of the part (Table 4, optimal choice: (c)/(d), 2218.3, 2054.8).

The best results were achieved for n = 9, m = 9, and 18 neurons in the hidden layer (i.e., MLP 5-18-10, Figure 3), but we observed that other ANN structures were also effective, such as MLP 5-10-10 and MLP 5-27-10.

ANN was able to minimize the MSE for the data in the training set to very small values (0.001–0.01) (Table 5 and Table 6, the best choice: MLP 5-18-10, 0.9529, 0.9712, 0.001).

ANN, unlike conventional modeling methods, allows the relatively simple and quick solution to the 3D-printing optimization problem without the need to define an algorithm/function, based only on ANN input and output variables. A key issue is the proper selection of data reflecting the actual dependence of the problem under investigation and the method of their processing in such a way that achieves appropriate correlations between the input and output variables. Thus, the key issue is not a relatively simple ANN, but proposing such a set of parameters, that will make up a model set of parameters for a 3D printer based on knowledge gleaned from previous experience in the printing of the exoskeleton parts. The goal of the AI-based optimization is not to find the optimum of the objective function, but to use it to determine the best set of 3D printing parameters in each case of a printed exoskeleton part.

## 5. Discussion

The automation of the process of selecting the 3D printing parameters requires a one-time step during the development of the proper tool (ANN). The long-term advantages of the above approach include both improved quality of the 3D printing and the reduced time required to prepare such a semi-automatically created set of parameters compared to the time that must be spent by an engineer each time. Our solution may be easily adapted to other material/printer combinations and developed to create a more universal tool, useful for Industry 4.0 purposes, thanks to quicker visualization of concepts, rapid prototyping, and a novel way for rapid manufacturing of patient-tailored medical equipment [35]. Our results confirm that the PLA- and PLA+-based 3D-printing process may be optimized toward the successful printing of usable/functional elements of an exoskeleton. We believe that the next step in the area of biomedical 3D printing is the development of specialized filaments adapted to suit professional biomedical applications. In our opinion, the 3D printing of elements for the needs of biomedicine is a complicated issue, and the materials, besides their specific mechanical properties, must be biocompatible, as confirmed by appropriate certificates [11]. The presence of an elevated temperature alters the biological properties of the material, or even make it impossible to use it in conjunction with living tissue, body fluids, etc. [36].

The choice of hardware (3D scanner, 3D printer) and software (modeling software, etc.) may significantly influence mitigation of the design, production costs, data accuracy, process efficiency, and multifunctionality of the project [37]. Easier modification through the use of modifiable templates and faster production achieved through direct 3D printing closer to the user home [38] are also of high importance as far as patient satisfaction is concerned [39]. Precise selection and fitting procedures may vary between patients (e.g., according to age, dimensions, weight, and strength), even between healthy people and those affected by various kinds and levels of deficit that compromise their upper limb functionality [40]. Such an orthosis may provide greater flexibility in hand movement and stronger support compared to a traditional, manually-formed orthosis [41]. Further steps should include research into the manufacturing processes required to create ready-to-use exoskeletons or orthosis parts that are carried out in a controlled environment, i.e., automatic design of patient-tailored objects based on the 3D-scanned geometry of a patient [42]. Individualized training program speeds up the fitting and learning time, while the parametric model shortens the complicated orthosis design process to a few minutes [43].

The mechanical, thermal and/or dimensional properties of the materials can also be unique and not achievable in traditional manufacturing, even with the simultaneous application of multi-material printing [44]. This situation may create a very good base for Industry 4.0 processes, due to:

- Integration of the physical and virtual processes thanks to the application of the software for large-scale machine production,
- automation,
- use of digital twins,
- reliability and continuous detection of errors,
- cost reduction,
- resultant increased efficiency thanks to manufacturing optimization with the simultaneous increase in the quality of the products.

It is difficult to discuss our findings in the context of the results achieved by other researchers in this area. There are only a few similar studies [41,42]. The results achieved in our work are comparable to those achieved by other authors in this area. Our study can be included among the first researches performed in the field by other researchers. For the first time, this research focuses on the optimization of materials used for three-dimensional printing of exoskeleton parts. It concerns such an advanced device made with the 3D printing technique as an exoskeleton, while the brace can be built in a much simpler way [41,42,43].

Future research directions include issues related to the required novel features (e.g., memory of shape) and materials (e.g., those created specially for 3D printing of biomedical products) [45]. Despite the relatively good levels of current knowledge and experience within 3D-printing, there are still many options for processes and improvements that must be taken into consideration, including creating many sub-technologies (multi-material 3D printing, memory of shape) and opportunities to enable novel and challenging product features [46]. Additional factors include accuracy and speed of production, which can be often reduced by paying particular attention to the quality of the final product. There is no doubt that future research should focus on multi-material technologies or materials where the parameters or functions change or combine with various structural parameters, e.g., depth, as in living tissue. Compartmental studies on various filaments such as the PLA and PLA+ covered in the paper, but Thermoplastic polyurethane elastomers (TPU) (i.e., a copolymer consisting of hard and soft segments, allowing the creation of 3D models that are flexible after printing) and BioFlex (a biodegradable elastomer with good elastic properties and high impact strength) are also possible options. Thus, the current study may be regarded as preliminary to further exploration of computational optimization of the printed object parameters and control systems based on multi-material 3D-printers.

The solutions here are intended for the professional user. However, certain 3D-printing processes may be simplified and made at home, such as in rural areas with resource-limited healthcare [47]. The high number of open-source repositories may create novel occupations for home-based improvements and inventions in many areas, from self-printed plastic mugs and spare parts for toys through relatively advanced orthoses and exoskeletons to the most advanced full-color objects made from composites and/or metals, and more.

To optimize parameters using ANN more efficiently, a genetic algorithm was used to reduce the number of variables. Moreover, the parameters read directly from the printer’s software, as defined by the authors, can be used to accelerate the optimization process. The application of fuzzy sets, especially ordered fuzzy numbers, constitutes another area that remains unexploited so far.

MLP and various other ANNs may meet the requirement of researchers optimizing 3D-printing processes, such as radial networks (RBF), Hamming networks (MAGNET), or even self-organizing Kohonen networks. However, as they require many more neurons, the required computational power and thus their cost-effect factor are not so favorable. There are future limits to compromise (e.g., between quality and price), but these should be tested in clinical applications

Due to the increasing numbers of advanced processes related to the production and material features, 3D-printed exoskeletons require computational models to fully estimate and understand their behavior made under different conditions (hospital, ambulatories, or home-based rehabilitation). In our opinion, the application of AI-based models will play an important role within software used in 3D printers and settings for reverse engineering, especially in biomedical applications.

## 6. Conclusions

This paper presents a model based on ANN and GA used to optimize the materials and features required of 3D-printed exoskeletons. From this study the following conclusions can be drawn:

- Optimization and automation of the process- and material-related factors improves access to the material properties of objects required in everyday clinical practice, such as the components of a hand exoskeleton described here.
- The network indicated PLA+ as the most appropriate choice of material reaching maximal tensile force of 2218.3 N for part with optimal weight.
- Relatively simple combined ANN/GA solutions create a quick and reliable second opinion system, useful in the processing and material optimization of 3D-printed medical devices. The selection of the number of neurons in the hidden layer affects the effectiveness in predicting the output set of 3D printing process parameters. Despite the complexity of the processes, the number of the parameters taken into consideration within the optimization process can be reduced.
- By creating different structures of the ANN model, it was possible to demonstrate the most effective prediction possibilities for the model MLP 5-18-10. The use of various measures produced the best prediction results, (R)MSE = 0.001.

## Figures and Tables

**Table 1:** Selected process parameters for three-dimensional (3D) printing using polylactic acid (PLA) and PLA+ (before optimization) (own work based on [32]).

Parameter	Value Range	
	PLA	PLA+
Density (g/cm^3^)	1.2–1.3	1.2–1.3
Diameter (mm)	2.85 ± 0.02	2.85 ± 0.02
Layer height (mm)	0.04–0.32	0.04–0.32
Shell thickness (mm)	≥0.15	≥0.15
Bottom thickness (mm)	≥1	≥1
Top thickness (mm)	≥1	≥1
Fill density (%)	10–100%	10–100%
Print speed (mm/s)	30–70	30–70
Bed temperature (°C)	50–60	50–60
Printing temperature (°C)	205–225	205–225
Maximum extrusion temperature (°C)	225	225
Printer power consumption (W)	80	80
Relative tensile strength	100	108.5
Relative bending strength	100	134.4
Relative modulus of elasticity in bending	100	114

**Table 2:** The best multi-layer perceptron (MLP) network models for diagnostic measures.

NS—ANN Structure	AH—Activation Function in the Hidden Layer	AO—Activation Function in the Output Layer
5-10-10	Sigmoid	Sigmoid
5-18-10	Sigmoid	Sigmoid
5-27-19	Sigmoid	Sigmoid

**Table 3:** Optimal parameters for 3D printing using PLA and PLA+.

Parameter	Optimal Value	
	PLA	PLA+
Layer height (mm)	0.2	0.2
Shell thickness (mm)	1.5	1.2
Bottom thickness (mm)	2	2
Top thickness (mm)	2	2
Fill density (%)	40	40
Print speed (mm/s)	35	70
Bed temperature (°C)	60	55
Printing temperature (°C)	220	215
Second nozzle temperature (°C)	220	220
Maximum tensile force—weak part (N)	1523.6	1640.2
Maximum tensile force—optimal part (N)	2054.8	2218.3
Maximum tensile force—strong but heavy part (N)	1988.7	2163.1

**Table 4:** Maximum tensile force for selected exoskeleton samples.

Sample Part of the Exoskeleton (PLA+/PLA)	Maximum Tensile Force for PLA+ (N)	Maximum Tensile Force for PLA (N)
(a)/(b)	1640.2	1523.6
(c)/(d)	2218.3	2054.8
(e)/(f)	2163.1	1988.7

**Table 5:** Selected ANNs quality assessment.

Network Name	Quality (Learning)	Quality (Testing)
MLP 5-10-10	0.8917	0.9189
MLP 5-18-10	0.9529	0.9712
MLP 5-27-10	0.9330	0.9545

**Table 6:** Root Mean Square Error (RMSE) values for three MLP neural network.

Network Name	RMSE
MLP 5-10-10	0.01
MLP 5-18-10	0.001
MLP 5-27-10	0.02
